# Supplementary figures and images for: Increased dopaminergic neurotransmission results in ethanol dependent sedative behaviors in Caenorhabditis elegans
Source: PLoS Genet. 2021 Feb 1;17(2):e1009346. doi: 10.1371/journal.pgen.1009346 (PMC7877767; doi:10.1371/journal.pgen.1009346)

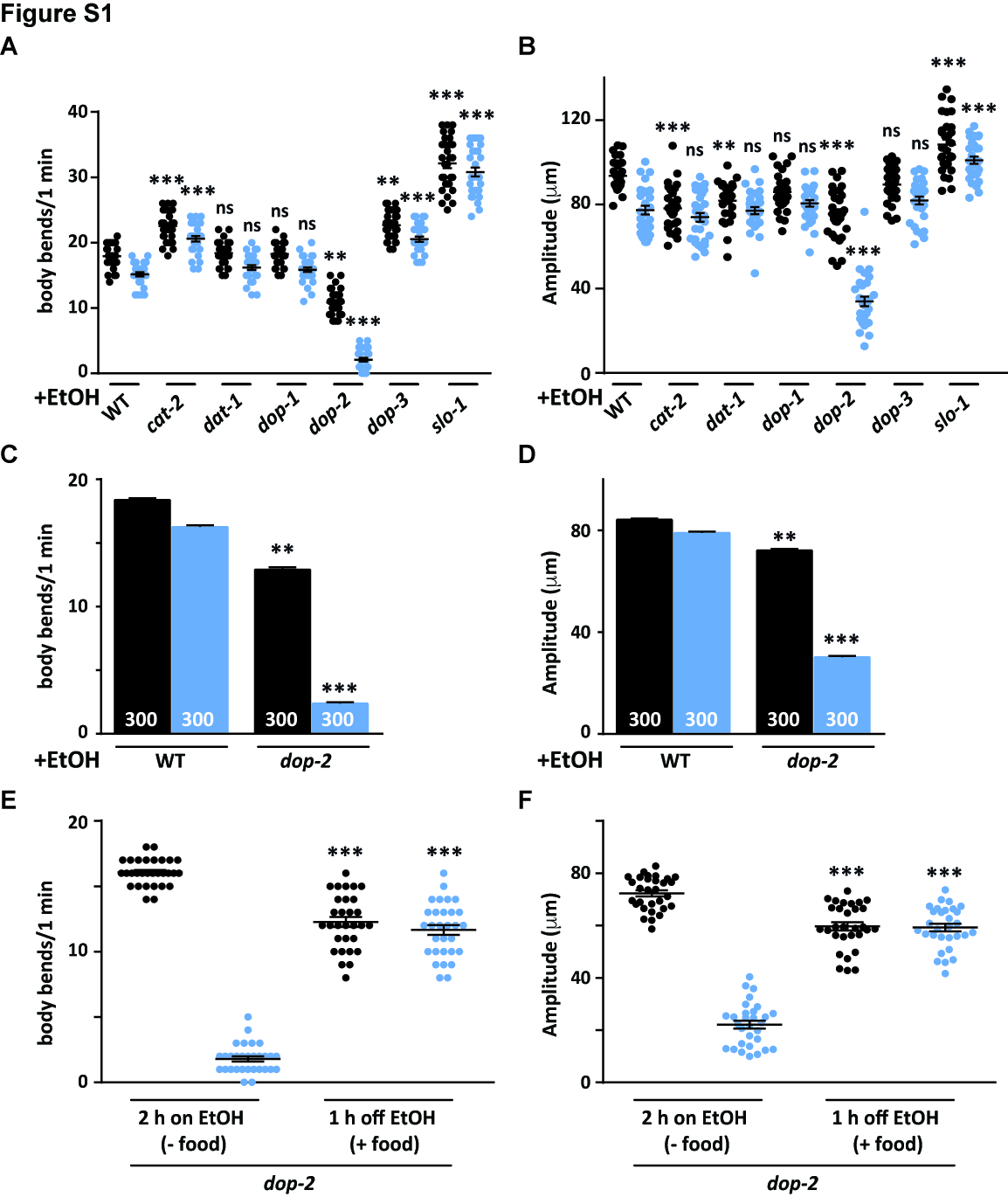

Supplement: S1 Fig — (A) Graph of number of body bends (anterior body bends are shown in black and posterior body bends are shown in blue in all graphs) in wild type (WT), cat-2, dat-1, dop-1, dop-2, dop-3 and slo-1 animals upon Ethanol (EtOH) treatment, (n = 10, N = 3 and F = 66.8, DF = 13). Please note that although we did not find a significant difference between WT and dop-1 animals in this experiment, we did however find a significant decrease in the number of body bends seen in dop-1 mutants that were outcrossed more times. These data are shown in Fig 5A. (B) Graph of amplitude of body bends (anterior amplitude of body bends are shown in black and posterior amplitude of body bends are shown in blue in all graphs) for WT, cat-2, dat-1, dop-1, dop-2, dop-3 and slo-1 mutants upon EtOH treatment, (n = 10, N = 3 and F = 82.2, DF = 13). (C) Graph of number of body bends plotted from pooled data of WT and dop-2 animals upon EtOH treatment, (n = 30, N = 10 and F = 1196, DF = 3). (D) Graph of amplitude of body bends from pooled data of WT and dop-2 animals upon EtOH treatment, (n = 30, N = 10 and F = 1920, DF = 3). Our data throughout this manuscript show differences in the values plotted for WT and dop-2 animals. In order to get a better understanding of all the data included throughout this manuscript, data from all experiments were pooled for WT and dop-2 mutants and depicted as a bar-graph in figures C and D. (E) Graph of number of body bends quantitated for dop-2 mutant animals under different conditions including 2 hours (hr) on EtOH (-food) and the same animals transferred to NGM plates with food to study the recovery of EIS behavior in dop-2 mutant animals, observed and quantitated after 1 hr on food, (n = 10, N = 3 and F = 406, DF = 3). (F) Graph of amplitude of body bends quantitated for dop-2 mutant animals under different conditions including 2 hr on EtOH (-food) and the same animals transferred to NGM plates with food to study the recovery of EIS behavior in dop-2 mutant anima [file pgen.1009346.s001.tif]

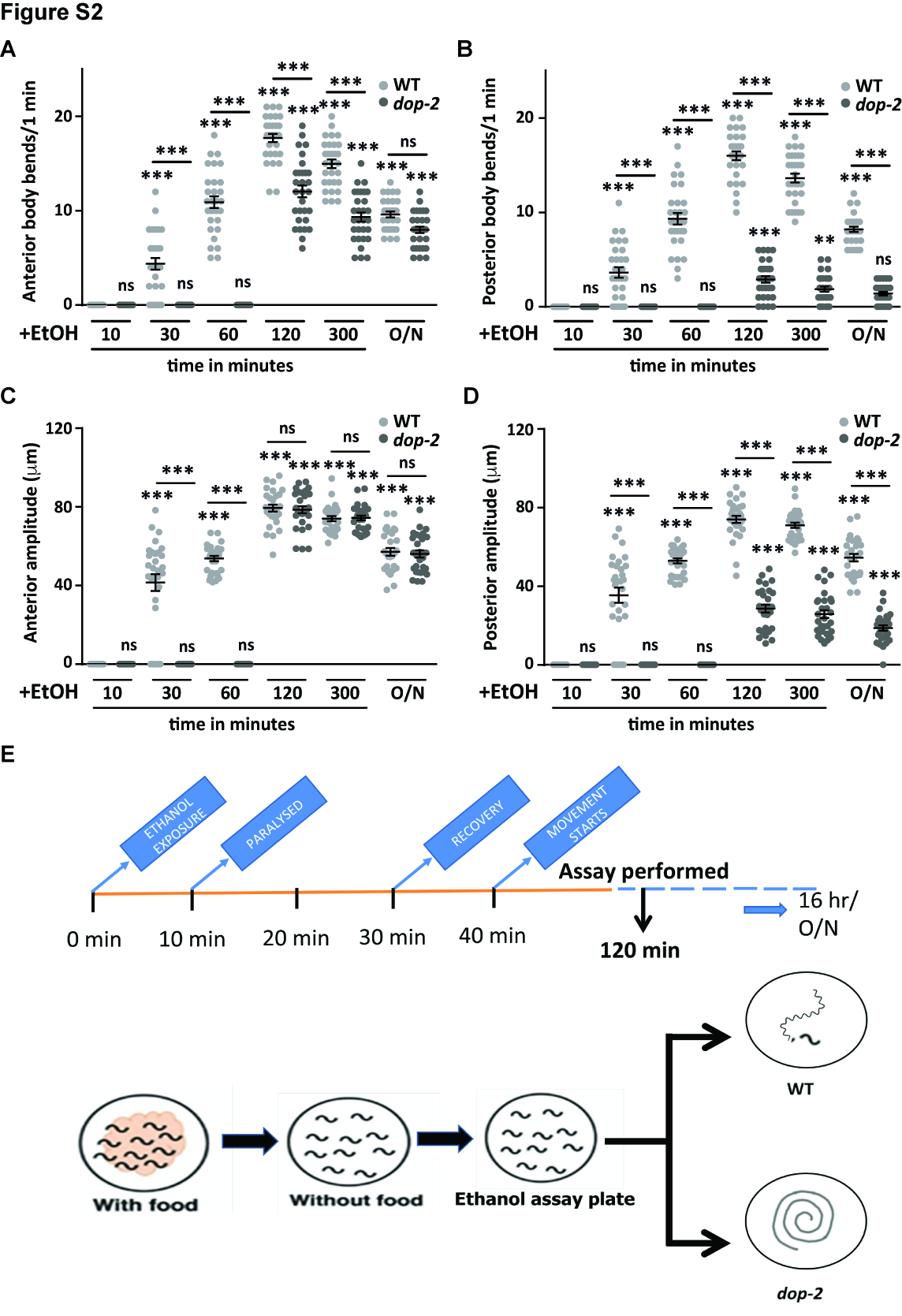

Supplement: S2 Fig — (A) Number of anterior body bends quantified from WT and dop-2 mutant upon EtOH treatment at 10 minutes (min), 30 min, 60 min, 120 min, 300 min and overnight (16 hr) time points (n = 10, N = 3 and F = 239, DF = 11). (B) Number of posterior body bends quantified from WT and dop-2 mutant animals during an EtOH assay at 10 min, 30 min, 60 min, 120 min, 300 min and overnight (16 hr) time points (n = 10, N = 3 and F = 272, DF = 11). (C) Amplitude of anterior body bends quantified from WT and dop-2 mutant animals upon EtOH treatment at 10 min, 30 min, 60 min, 120 min, 300 min and overnight (16 hr) time points (n = 10, N = 3 and F = 372, DF = 11). (D) Amplitude of posterior body bends quantified from WT and dop-2 mutant animals upon EtOH treatment at 10 min, 30 min, 60 min, 120 min, 300 min and overnight (16 hr) time points (n = 10, N = 3 and F = 263, DF = 11). (E) Diagrammatic representation of the EtOH assay over time (this image is also shown in Fig 1A) and a representation of WT and dop-2 EIS phenotypes on EtOH assay plates (bottom right). The same videos of moving animals were used to quantitate both number of body bends and the amplitude of body bends for each genotype. Error bars represent ±S.E.M., “n” represents the number of animals and “N” represents the number of replicates. The p-values were calculated using one-way ANOVA and Tukey-Kramer multiple comparison test; “**” indicates p<0.01, “***” indicates p<0.001 and “ns” indicates not significant in both graphs. For all graphs the statistical comparison right above each genotype indicates a comparison with the WT control animals at 10 min post EtOH treatment. Other statistical comparisons are indicated above lines indicating the genotypes that are being compared. (TIF) [file pgen.1009346.s002.tif]

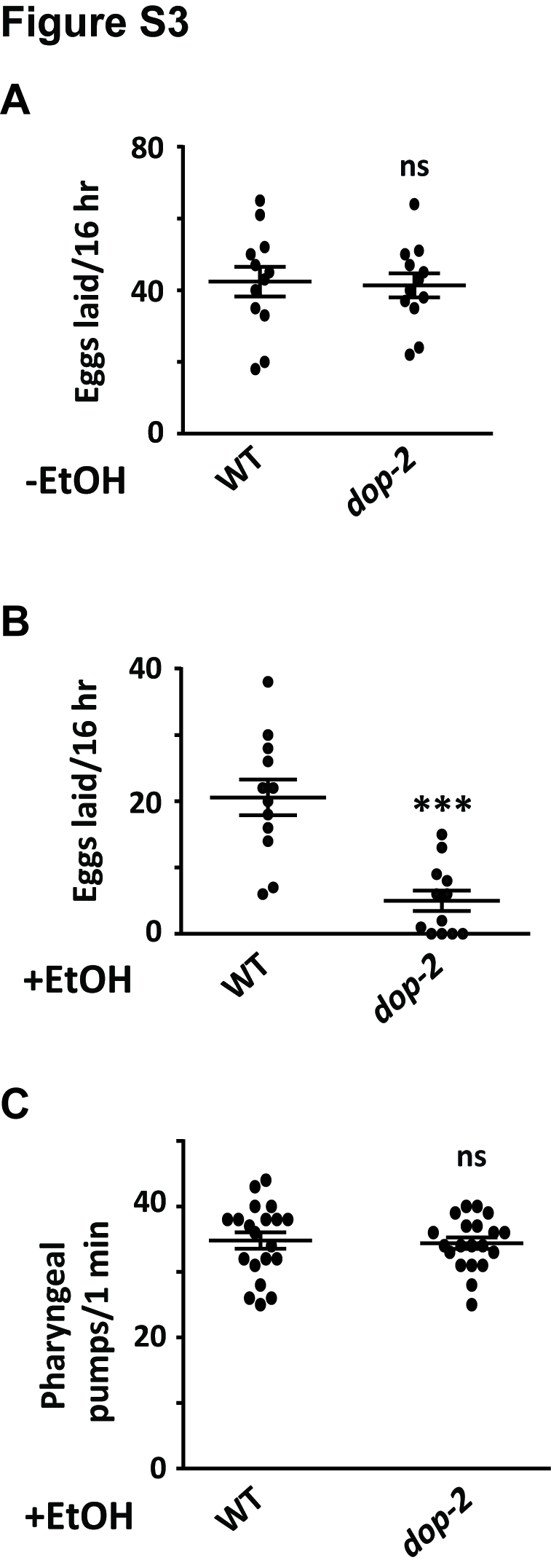

Supplement: S3 Fig — (A) Graph depicting the number of eggs laid over a 16 hr time period for WT and dop-2 animals. The assay was performed on plates with food and without EtOH. The data was plotted from 12 animals for both genotypes (t = 0.2030 and df = 21.04). (B) Graph depicting the number of eggs laid over a 16 hr time period from WT and dop-2 animals. These assays were performed on plates without food and with EtOH. The data was plotted from 12 animals for both genotypes (t = 0.2030 and df = 21.04). (C) Number of pharyngeal pumps per min, recorded from WT and dop-2 animals after 120 min of EtOH treatment. The data were plotted from 20 WT and 20 dop-2 animals, (t = 0.26 and df = 34.05). Error bars represent ±S.E.M. and p-values were calculated using t-test with Welch’s correction; “***” indicates p<0.001 and “ns” indicates not significant. (TIF) [file pgen.1009346.s003.tif]

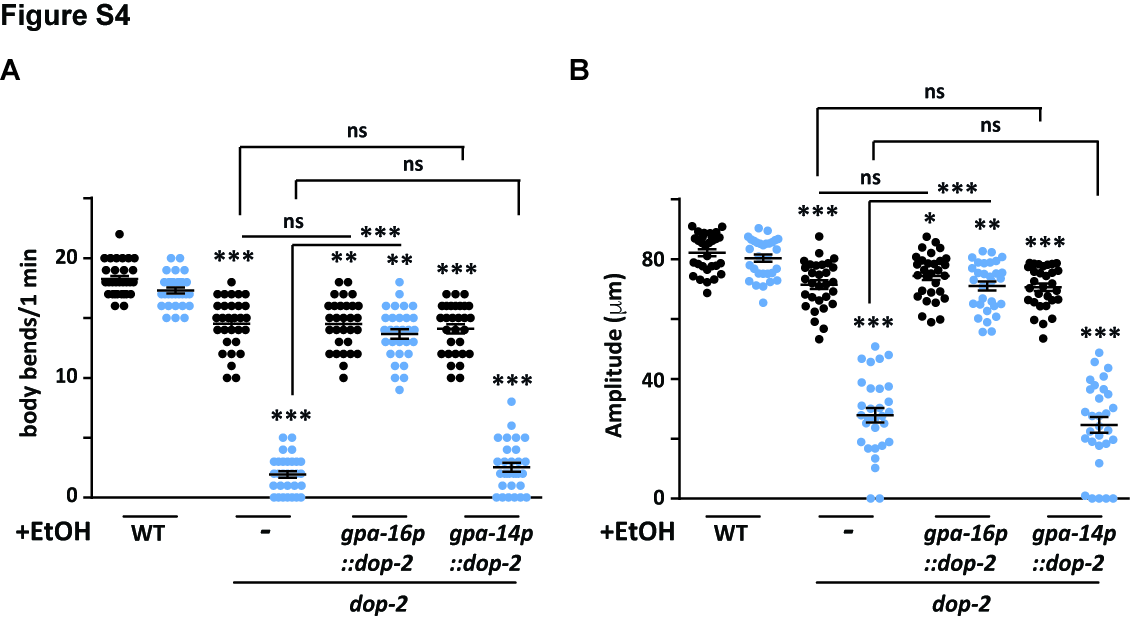

Supplement: S4 Fig — (A) Graph representing the number of body bends quantitated from WT, dop-2, dop-2; gpa-16p::dop-2 and dop-2; gpa-14p::dop-2 lines. Anterior body bends are shown in black and posterior body bends are shown in blue in the graph (n = 10, N = 3 and F = 336, DF = 7). (B) Graph representing amplitude of body bends quantitated from WT, dop-2, dop-2; gpa-16p::dop-2 and dop-2; gpa-14p::dop-2 lines. Anterior amplitude of body bends are shown in black and posterior amplitude of body bends are shown in blue the graph (n = 10, N = 3 and F = 181, DF = 7). The same videos of moving animals were used to quantitate both number of body bends and the amplitude of body bends for each genotype. Error bars represent ±S.E.M., “n” represents the number of animals and “N” represents the number of replicates. The p-values were calculated using one-way ANOVA and Tukey-Kramer multiple comparison test; “**” indicates p<0.01, “***” indicates p<0.001 and “ns” indicates not significant in both graphs. For both graphs the statistical comparison right above each genotype indicates a comparison with the respective WT control animals. Other statistical comparisons are indicated above lines indicating comparisons with dop-2 mutants. (TIF) [file pgen.1009346.s004.tif]

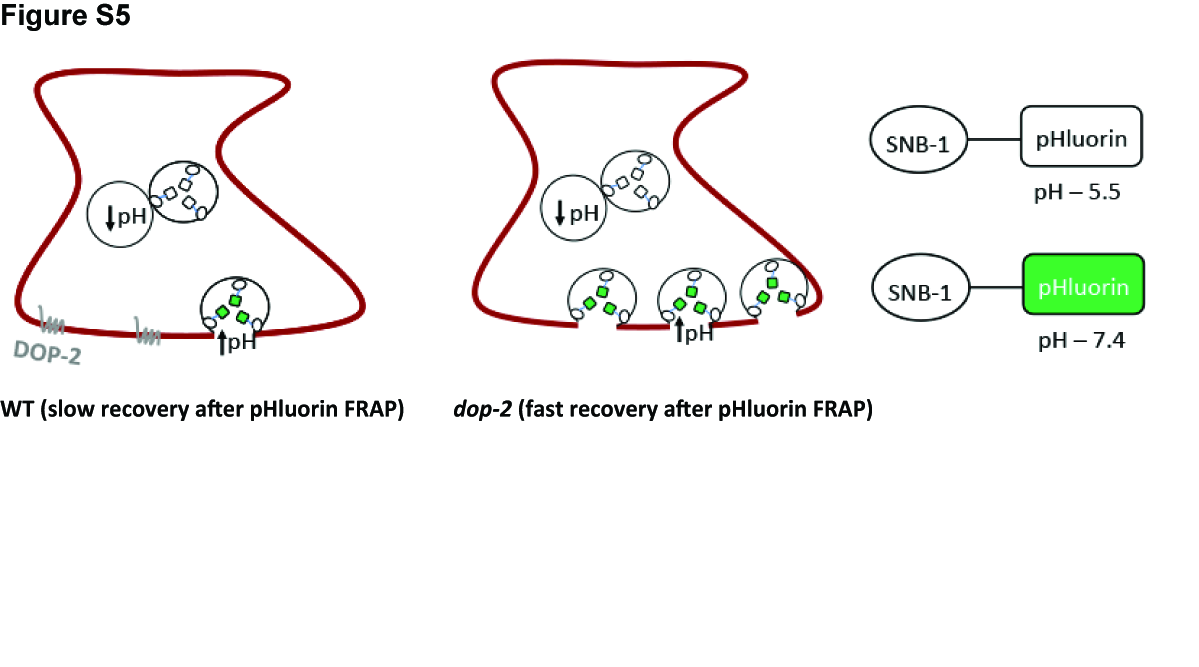

Supplement: S5 Fig — A pH sensitive GFP Fluorophore, pHluorin, was tagged to the synaptic vesicle protein SNB-1 and expressed in DA neurons. Caenorhabditis elegans lines expressing the pHluorin construct were imaged to quantitate dopamine release. This construct was previously used in [60]. (TIF) [file pgen.1009346.s005.tif]

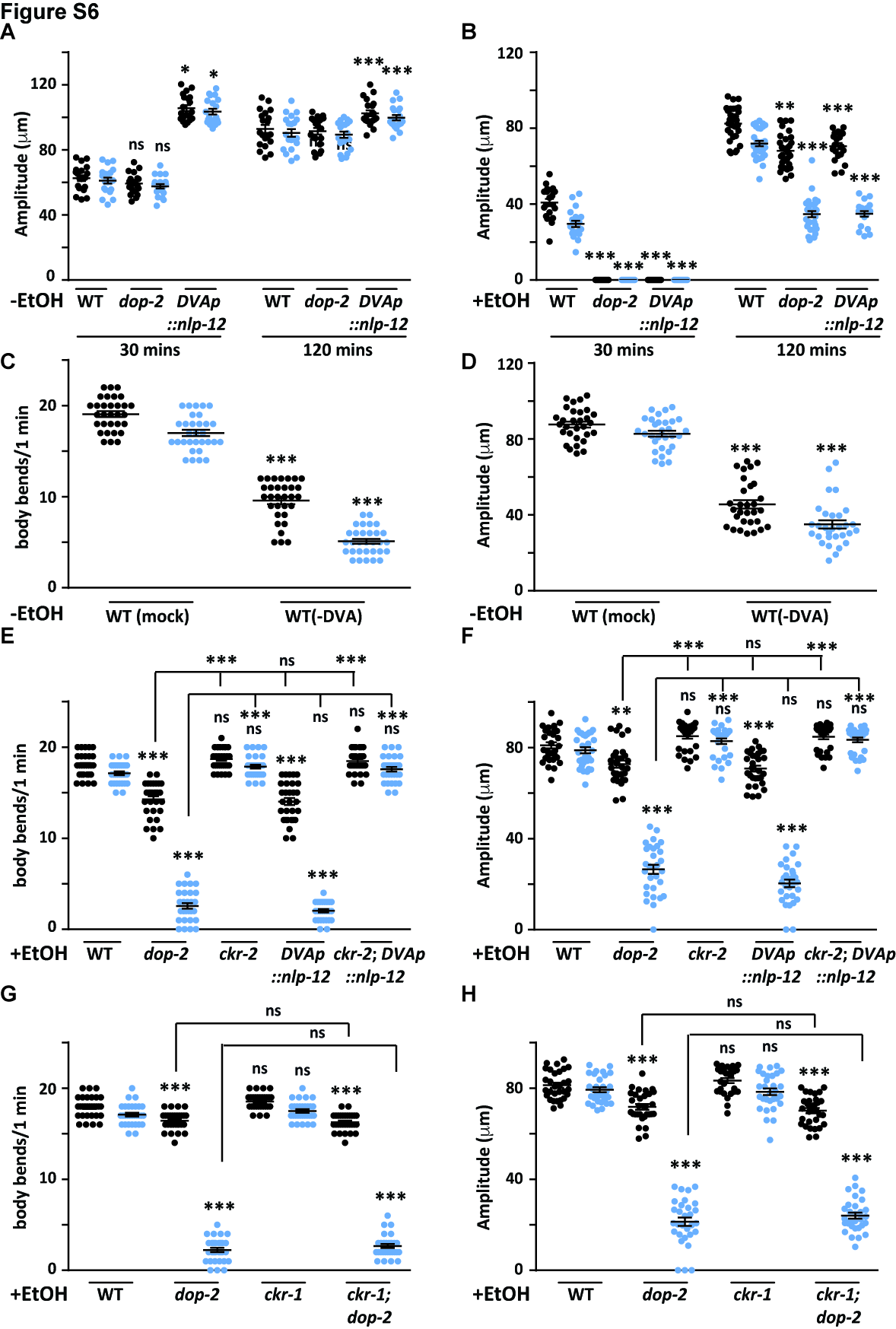

Supplement: S6 Fig — (A) Quantitation of the amplitude of body bends (anterior body bends are shown in black and posterior body bends are shown in blue in all graphs) from WT, dop-2 mutant animals and an NLP-12 overexpression line where NLP-12 is expressed in DVA neurons. These experiments were performed at 30 and 120 min off food without EtOH treatment, (n = 10, N = 2 and F = 104, DF = 11). (B) Quantitation of the amplitude of body bends (anterior amplitude of body bends are shown in black and posterior amplitude of body bends are shown in blue in all graphs) from WT, dop-2 mutant animals and an NLP-12 overexpression line where NLP-12 is expressed in DVA neurons. These experiments were performed at 30 and 120 min off food with EtOH treatment, (n = 10, N = 2 and F = 522, DF = 11). (C) Graph representing the number of body bends in mock and DVA ablated WT animals not treated with EtOH, (n = 10, N = 3 and F = 368, DF = 3). (D) Graph representing the amplitude of body bends in mock and DVA ablated WT animals not treated with EtOH, (n = 10, N = 3 and F = 192, DF = 3). (E) Graph representing the number of body bends quantitated from WT, dop-2, ckr-2, DVAp::nlp-12 and ckr-2; DVAp::nlp-12 treated with EtOH, (n = 10,N = 3 and F = 576, DF = 9). (F) Graph representing the amplitude of body bends quantitated from WT, dop-2, ckr-2, DVAp::nlp-12 and ckr-2; DVAp::nlp-12 animals treated with EtOH, (n = 10,N = 3 and F = 306, DF = 9). (G) Graph representing number of body bends quantitated from WT, dop-2, ckr-1 and ckr-1; dop-2 animals treated with EtOH, (n = 10,N = 3 and F = 1193, DF = 7). (H) Graph representing amplitude of body bends quantitated from WT, dop-2, ckr-1 and ckr-1; dop-2 animals treated with EtOH, (n = 10,N = 3 and F = 383, DF = 7). The same videos of moving animals were used to quantitate both number of body bends and the amplitude of body bends for each genotype. Error bars represent ±S.E.M., “n” represents the number of animals and “N” represents the number of replicates. The p-values [file pgen.1009346.s006.tif]

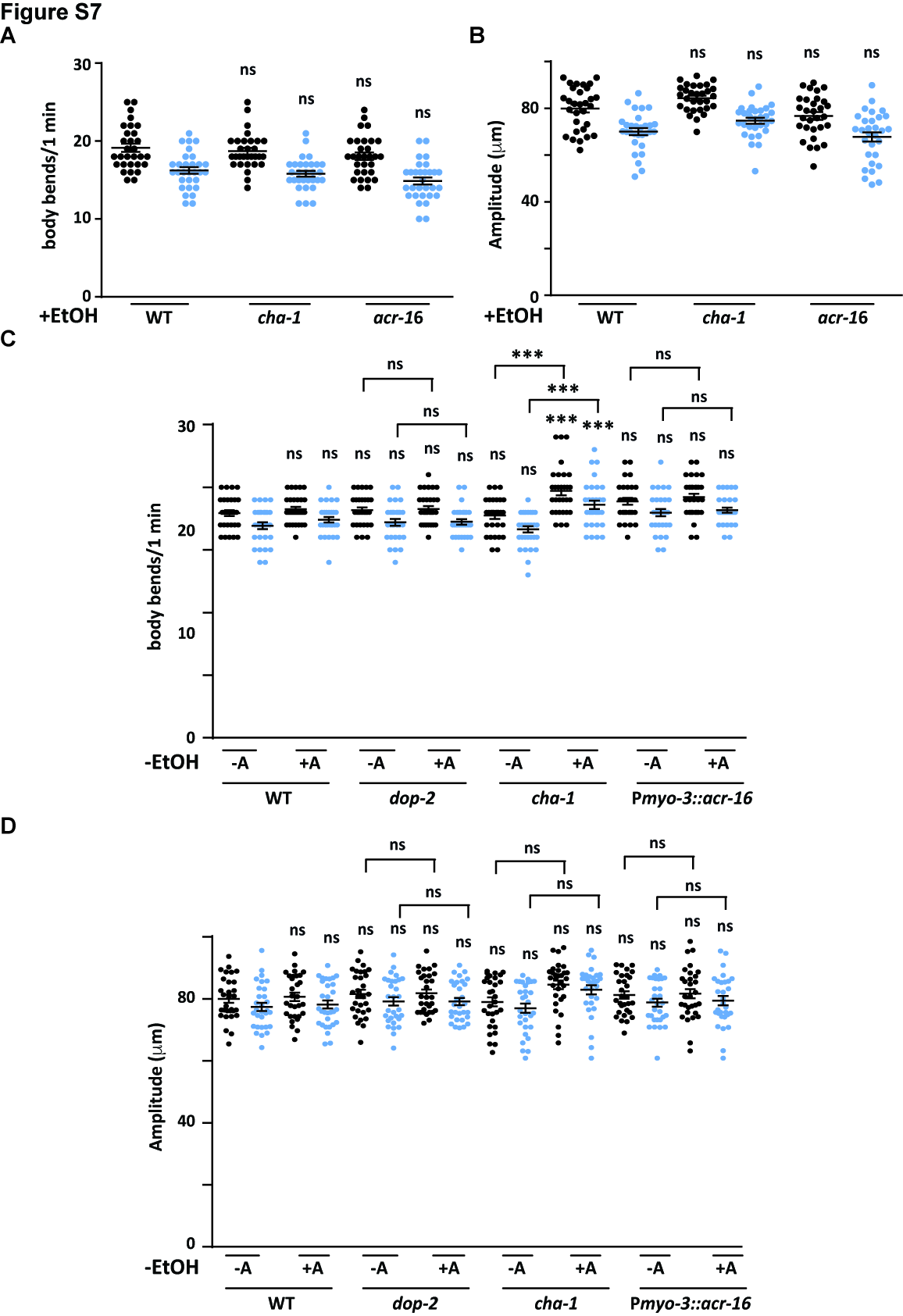

Supplement: S7 Fig — (A) Graph indicating the number of body bends quantitated from WT, cha-1 and acr-16 mutants upon EtOH treatment (n = 10, N = 3 and F = 15.0, DF = 5). (B) Graph indicating amplitude of body bends quantitated from WT, cha-1 and acr-16 mutants upon EtOH treatment (n = 10, N = 3 and F = 10.8, DF = 5). (C) Graph indicating the number of body bends quantitated from WT, dop-2, cha-1 and myo-3p::acr-16 under with aldicarb (+A) and without aldicarb (-A) conditions (n = 10, N = 3 and F = 10.1, DF = 15). (D) Graph indicating amplitude of body bends quantitated from WT, dop-2, cha-1 and myo-3p::acr-16 under with aldicarb (+A) and without aldicarb (-A) conditions (n = 10, N = 3 and F = 2.30, DF = 15). The same videos of moving animals were used to quantitate both number of body bends and the amplitude of body bends for each genotype. Error bars represent ±S.E.M., “n” represents the number of animals and “N” represents the number of replicates. The p-values were calculated using one-way ANOVA and Tukey-Kramer multiple comparison test; “ns” indicates not significant in all graphs. For both graphs the statistical comparison right above each genotype indicates a comparison with the respective WT control. Other statistical comparisons are indicated above lines indicating the genotypes that are being compared. (TIF) [file pgen.1009346.s007.tif]
