# Supplementary material for: Increased dopaminergic neurotransmission results in ethanol dependent sedative behaviors in Caenorhabditis elegans
Source: PLoS Genet. 2021 Feb 1;17(2):e1009346. doi: 10.1371/journal.pgen.1009346 (PMC7877767; doi:10.1371/journal.pgen.1009346)
Supplement: S1 Text — Includes Tables A, B and C that contain the complete list of strains, primers and plasmids used in this study. (DOCX) [file pgen.1009346.s019.docx]

**Supporting tables**

**Table A: List of strains used in this study**

| **Strain** | **Genotype** | **Comments** |
| --- | --- | --- |
| **BAB912** | *dop-2(vs105)V 4X* outcrossed (original strain LX702) | CGC strain |
| **BAB913** | *dop-3(vs106)X 2X* outcrossed (original strain LX703) | CGC strain |
| **BAB914** | *dop-1(vs100)X 2X* outcrossed (original strain LX645) | CGC strain |
| **BAB915** | *cat-2(n4547)II 3X* outcrossed (original strain MT15620) | CGC strain |
| **PR1152** | *cha-1(p1152)IV (outcrossed 4X)* | CGC strain |
| **BAB916** | *acr-16(ok789)V 2X* outcrossed (original strain RB918) | CGC strain |
| **BAB917** | *slo-1(eg142)V 2X* outcrossed (original strain BZ142) | CGC strain |
| **BAB918** | *dat-1(ok157)III 3X* outcrossed (original strain RM2702) | CGC strain |
| **LSC32** | *ckr-2(tm3082)III (outcrossed 8X)* | CGC strain |
| **BAB920** | *ckr-1(ok2502)I 2X* outcrossed (original strain RB1923) | CGC strain |
| **BAB919** | *nlp-12(ok335) 3X* outcrossed (original strain RB607) | CGC strain |
| **nuIs299** | *myo-3p*::*acr-16::gfp* | Josh Kaplan Lab |
| **IR724** | *asic-1*::*snb-1*::SEpHlourin | Blakely Lab |
| **BY834** | *dat-1p*::*dat-1::gfp* | Blakely Lab |
| **BZ555** | egls1(*dat-1p*::gfp) | CGC strain |
| **BAB900** | *dop-1(vs100); dop-2(vs105)* | This study |
| **BAB901** | *cat-2(n4547); dop-2(vs105)* | This study |
| **BAB902** | *egls1*(*dat-1p::gfp*); *dop-2(vs105)* | This study |
| **BAB903** | *dat-1p*::*dat-1*::GFP; *dop-2(vs105)* | This study |
| **BAB904** | *asic-1*::*snb-1*::SEpHlourin; *dop-2(vs105)* | This study |
| **BAB905** | *dop-2p*::DOP-2::CFP;*dop-2* (IndEx905) | This study |
| **BAB906** | *nlp-12p*::*nlp-12* (IndEx906) | This study |
| **BAB907** | *nlp-12p*::*dop-1*::*wrm*Scarlet; *dop-1(vs100); dop-2(vs105)* (IndEx907) | This study |
| **BAB908** | *nlp-12p*::*dop-1*::*wrm*Scarlet; *dop-1(vs100)* (IndEx908) | This study |
| **BAB909** | *myo-3p*::*acr-16::gfp*; *cat-2(n4547)* | This study |
| **BAB910** | *cha-1(p1152); dop-2(vs105)* | This study |
| **BAB911** | *ckr-2(tm3082); dop-2(vs105)* | This study |
| **BAB921** | *ckr-1(ok2502); dop-2(vs105)* | This study |
| **BAB922** | *ckr-2(tm3082);* *nlp-12p*::NLP-12 (IndEx906) | This study |
| **BAB923** | *gpa-16p::*DOP-2*; dop-2* (IndEx909) | This study |
| **BAB924** | *gpa-14p::*DOP-2*; dop-2* (IndEx910) | This study |
| **BAB925** | *nlp-12(ok335); dop-2(vs105)* | This study |

**Table B: List of primers used in this study**

| **Primer Code** | **Sequence** | **Comment** | **Gene** |
| --- | --- | --- | --- |
| **PRS37** | CCCTTGAATGGCCTCCACC | Genotyping Forward External | *dop-2* |
| **PRS38** | CAGTACTCCGGTACCGAGCAC | Genotyping Forward  Internal | *dop-2* |
| **PRS39** | CTCGGGAGCACTTGTGAGAG | Genotyping Reverse External | *dop-2* |
| **PRS23** | TCACAGATGTCCGTTTTCCA | Genotyping Forward External | *acr-16* |
| **PRS24** | TCAATGATTCCGAGTGACGA | Genotyping Reverse External | *acr-16* |
| **PRS 314** | GTGCCTGGAGGAGCGCAAATATTGG | Genotyping WT Forward | *slo-1* |
| **PRS 315** | GTGCCTGGAGGAGCGCAAATATTAA | Genotyping Mutant Forward | *slo-1* |
| **PRS 316** | GGACTTGCCCTGCGGTCCCGAATAC | Genotyping Reverse | *slo-1* |
| **PRS 322** | CCAAATTAGTCGAAAAGCTGATCCCGC | Genotyping Forward External | *dat-1* |
| **PRS 323** | GTGATCCTTGCCTGGGGGCTTC | Genotyping Forward Internal | *dat-1* |
| **PRS 324** | GAAGCCCCCAGGCAAGGATCAC | Genotyping Reverse External | *dat-1* |
| **PRS 332** | GGAATAGGAACCATAGAAGATCTCC | Genotyping Forward External | *cat-2* |
| **PRS 333** | CGATGACTGTGACACCGCGAGG | Genotyping Reverse External | *cat-2* |
| **PRS 334** | GGCCGAGAACTGATAACCCAGC | Genotyping Reverse Internal | *cat-2* |
| **PRS 340** | GGACCCAAACATGCCACAGTGATATGG | Genotyping Forward External | *dop-1* |
| **PRS 341** | GAAGATTCAGGCGAGTTGCATTCGC | Genotyping Reverse External | *dop-1* |
| **PRS 342** | GAATGCTCGTCTAAAGTCACGATTG | Genotyping Forward Internal | *dop-1* |
| **PRS 343** | GGTGTTCGCAATATTTGCGAAGACG | Genotyping Forward External | *dop-3* |
| **PRS 344** | CCATCAGCGTGCTTTACTCGTTCAC | Genotyping Reverse External | *dop-3* |
| **PRS 345** | GTGACGGTTTGTAGAGATCGTTCTC | Genotyping Forward Internal | *dop-3* |
| **PRS587** | GTGTGCCGAGAAAAGTCCACTG | Genotyping Forward | *cha-1* |
| **PRS588** | CTCGATCCGGTTGAATATTGTACG | Genotyping WT Reverse | *cha-1* |
| **PRS588** | CTCGATCCGGTTGAATATTGTAAA | Genotyping Mutant  Reverse | *cha-1* |
| **AS7** | ACCTGGCGGGGGAAACAACA | Genotyping Forward External | *ckr-2* |
| **AS8** | CCGTCAGCGAAATCTATCGT | Genotyping Reverse External | *ckr-2* |
| **AS5** | CACTCCATCACTACGACAGT | Genotyping Forward Internal | *ckr-2* |
| **PRS 473** | CCCCCCGGGATGAACGATTTGCAATGGCC | Cloning Forward XmaI | *dop-1* cDNA |
| **PRS 474** | CCCCCCGGGCTATTCCGGAATGGTTTCCTCG | Cloning Reverse KpnI | *dop-1* cDNA |
| **AS1** | AACTGCAGGGCCGAGACGAATCCGGAGG | Cloning Forward PstI | *nlp-12p* |
| **AS4** | CGGGATCCGCATTTTGTCGGAGGCAATTG | Cloning Reverse BamHI | *nlp-12p* |
| **AS3** | CGGGATCCGAAAATGTGTCGCTTCGAGAC | Cloning Reverse BamHI | NLP-12 |
| **PRS 605** | ACATGCATGCCCTCTCTTCGTCTTCTTCTTC | Cloning Forward SphI | *gpa-14p* |
| **PRS606** | CCCCCCGGGCTCAACTATAATGTCCTGAAATAC | Cloning Reverse XmaI | *gpa-14p* |
| **PRS607** | \| ACATGCATGCGATTCTTAGCATTTTTGCGG \| \| --- \| | Cloning Forward SphI | *gpa-16p* |
| **PRS608** | CCCCCCGGGCTGCAAAAAAATTTGGGATTTTTAGC | Cloning Reverse XmaI | *gpa-16p* |
| **PRS609** | CAGTATCTTATTCCAGAGCATCG | Genotyping Forward External | *ckr-1* |
| **PRS610** | GAGAATTAATTGGGAGGATGAAC | Genotyping Reverse External | *ckr-1* |
| **PRS611** | CACGTGTGACTCCACGAAAG | Genotyping Forward Internal | *ckr-1* |
| **PRS317** | AGAGACATGGAATGTCTCCC | Cloning Forward  NheI | *dop-2* |
| **PRS318** | GGTGGTAAAGTCATTGTTCGTCCGCCTAC | Cloning Reverse  XmaI | *dop-2* |

**Table C: List of plasmids used in this study**

| **S. No.** | **Plasmid No.** | **Plasmid** | **Source** |
| --- | --- | --- | --- |
| 1 | pBAB911 | *dop-2p::dop-2::cfp* | Rene Garcia Lab |
| 2 | pBAB912 | *nlp-12p*::GFP | This Study |
| 3 | pBAB913 | *nlp-12p*::NLP-12 | This Study |
| 4 | pBAB914 | *nlp-12p*::*sl2*::*wrm*Scarlet | This Study |
| 5 | pBAB915 | *nlp-12p::dop-1::* sl2::*wrm*Scarlet | This Study |
| 6 | pBAB916 | *gpa-14p::dop-2* | This Study |
| 7 | pBAB917 | *gpa-16p::dop-2* | This Study |
