# Supplementary material for: Increased dopaminergic neurotransmission results in ethanol dependent sedative behaviors in Caenorhabditis elegans
Source: PLoS Genet. 2021 Feb 1;17(2):e1009346. doi: 10.1371/journal.pgen.1009346 (PMC7877767; doi:10.1371/journal.pgen.1009346)
Supplement: Attachment — Submitted filename: Pandey, Singh et al Response to Reviewers.pdf [file pgen.1009346.s020.pdf]

Overview:

We thank the Associate Editor, Liliane Schoofs, the Editor-in-chief, Gregory P. Copenhaver and the Reviewers' for their helpful comments that we believe have greatly improved our manuscript and we have tried our best to respond to the points raised by them.

The Reviewers' have brought up some good points and we appreciate the opportunity to improve the presentation of our data to make it more convincing.

In response to the Reviewers' comments, regarding the timeline of recovery of wild-type and *dop-2* mutants, we have performed a time-course for recover from 10 minutes to 16 hours with multiple time points and have added that data to our manuscript. The Reviewers also wanted us to strengthen the section on the function of *dop-2* in PDE neurons. Unfortunately, we were unable to get a PDE specific promoter. However, we did perform *dop-2* rescue experiments with the *gpa-14* promoter (expressed in a subset of neurons including the anterior dopaminergic ADE neurons but not the posterior PDE) and the *gpa-16* promoter (expressed in a subset of neurons including the posterior PDE neurons but not the anterior dopaminergic neurons). Data from these experiments further strengthen our argument that *dop-2* is likely required in the PDE neuron to rescue the Ethanol Induced Sedative (EIS) phenotype seen in the mutants.

As suggested by the Reviewers', we have performed additional experiments to better understand the role of DOP-2 in the circuit involving NLP-12 and CKR-2. We have found that *ckr-2* suppresses the EIS phenotype seen in the NLP-12 overexpression line and mutants in *ckr-2* but not *ckr-1* suppress the *dop-2* EIS phenotype.

Taken together, these new results strongly suggest a role for *dop-2* and dopamine in maintaining locomotion in the presence of ethanol. Moreover, the mechanism of DOP-2 functioning in ethanol appears to be through the NLP-12 neuropeptide in the DVA neuron and its receptor CKR-2 that together allow for normal locomotion through motor neurons and the body-wall muscles.

These results as well as the complete response to the Reviewers' comments are detailed below. The response to each comment and the changes made to the manuscript are indicated in blue. We have gone through the comments very carefully, and are now submitting a revised manuscript that we believe takes into consideration all the concerns of the Reviewers'. We hope that the Editor and Reviewers' agree that these changes have addressed their concerns and that our manuscript is now acceptable for publication in *PLoS Genetics*.

Reviewer #1

In this study, Pandey et al. describe an ethanol-induced sedative behavior which occurs due to increased dopamine signaling by the PDE sensory neurons. They propose a model where dopamine activates the DVA neuron, promoting the release of NLP-12 neuropeptides which in turn activates cholinergic motor neurons. This causes a hypercontracted state and the authors suggest this is the cause of the sedation. This circuitry tested in this study was mostly previously described here: Davies et al 2003, Hawkings et al. 2015, Hu et al 2011). However, in the current study, EIS is being proposed as a novel behavior and the authors describe a new role for DOP-2.

I do have some significant concerns about the description of EIS, rationale and conclusions of the study.

We thank the Reviewer for their comments and concerns. We have addressed each point raised by the Reviewer below:

1. The authors describe a "novel locomotory phenotype" when animals are exposed to 400mM EtOH. However, it appears that this behavior is largely artificial. It only occurs in the presence of non-physiological levels of dopamine (i.e. in the absence of dop-2 or upon the application of dopamine). It seems that a more accurate description would be that heightened dopamine inhibits recovery from acute ethanol exposure. In other words, the increased dopamine is just prolonging the activation of the circuit PDE -> DVA -> motor neurons which has been previously described (Hawkins et al. 2015).

We thank the Reviewer for this comment. We agree that the behaviour seen is artificial in the presence of non-physiological levels of dopamine. We have replaced "novel locomotory phenotype" with "aberrant locomotory phenotype" in line 125. We have also added the Hawkins et al., 2015 reference here (line 127).

2. The authors argue that this is a new behavior but the description of it is undeveloped. The only quantifiable components of this new behavior is 1 minute of body bending counts and a single measurement of body amplitude 120 minutes after ethanol exposure. Based on previous studies, ethanol is known to depress locomotion. In fact, in Davies 2003, they measure speed over time. It is unclear why this behavior is novel based on the way it is described. A temporal description of the dop-2 animal's behavior compared to WT would make this more descriptive and useful. This would allow for other means of comparisons between genotypes and future studies. The authors explain what they qualitatively observed of the dop-2 animals between 0-120 minutes but do not show quantitative data. If EIS is novel, then a better description should be provided. Some of this is talked about in the methods section as well, but the data is not shown and it is unclear why it is in the methods section.

We thank the reviewers for this comment that this is not a novel behaviour and for the suggestion that we need to provide a time paradigm based explanation for our

behaviour. We have changed the word novel to aberrant (line 125, please see above point). We have also performed and quantitated the ethanol dependent behaviour over a longer period of time at 10 min, 30 min, 60 min, 120 min, 300 min and overnight ie (16 hour) time points. These data have been added to the manuscript in lines 177-186 in the results section, lines 589-594 in the methods section and in Figure S2.

3. EIS reversibility is never quantified but just mentioned in the methods. Do the animals recover? If so, when? Are the animals just dying prematurely? Can they be rescued if removed from ethanol? Where is the data for this?

Thank you for this comment. We agree that showing recovery/death of the animals after exposure to EtOH is important. Briefly, the animals do recover when removed from EtOH and added to a plate with food. The animals do not die prematurely. As suggested we have analysed the recovery and plotted the data for body bends and amplitude after one hour of recovery for the *dop-2* mutant animals as shown in Figure S1e, f and in lines 594-595 in the methods section.

4. The conclusions on Lines 183 and 210 about EIS being a dop-2 dependent behavior are not correct. EIS only occurs in the absence of dop-2. It would be more accurate to suggest that EIS is induced by dopamine and a lack of dopamine signaling suppresses EIS.

Thank you for this comment we have accordingly changed the wording in these two lines to indicate that EIS happens in the absence of *dop-2*. This is updated in lines 207 and 244-245.

5. Overall, the conclusions throughout the manuscript are hard to follow the way they are worded.

We have reworded the conclusions throughout the manuscript.

Some more minor concerns:

1. The authors provide a list of movies, however, the movies would benefit by having captions added to them.

We apologise for this oversight. We have added captions to all the movies.

2. Movie 5 – Animals look like rollers. Is the roller phenotype something that occurs with EIS and if so, can you discuss? The idea of them moving in circles is mentioned, is this related?

We have changed this movie, the behaviour of the EIS animals is different from the roller phenotype. In the case of *roller* phenotype, the animals roll at one place, while in the EIS phenotype the *C. elegans* move in larger circles, at times covering the whole 60 mm plate diameter. We specifically find circles made by EIS animals as compared to the very small and dispersed circles made by *roller* animals. The plate phenotype for EIS animals is shown in Fig. 1g and elaborated in lines 163-165.

3. Supplementary figure 3 was skipped or numbering was off.

Thank you for pointing this out and we apologise for the oversight. We have now changed the supplementary figure numbers to sequential numbers from Figures S1-S7 (total 7 supplementary figures).

4. Line 92 – "…function through the G-protein signaling pathway." This seems too vague, can you be more specific about which one and how? This seems relevant because the model predicts that these pathways are employed to increase neuropeptide release.

The line here deals with general functioning of dopamine receptors through their activation of G-protein signaling. We have however added a line for G-proteins (lines 90-96). In *C. elegans*, it has been reported that DOP-2 physically interacts with GPA-14 (Gα) protein (Pandey and Harbinder 2012) in the ADE neuron. Hence, it is likely that DOP-2 functions in a similar manner in other neurons.

5. Line 99 – "….been associated with alcoholism." It would be interesting to add a sentence that explains how they are associated, considering this is a major connection for this study.

Thank you for this suggestion. We have added the below sentence (lines 101-106): "Studies have reported a correlation between D2 receptors and alcohol consumption and the development of alcoholism Lu et al., 2001; Thanos et al., 2005; Volkow et al., 2006; Kraschewski et al., 2009). Further, studies have also shown that D2 receptor levels are enhanced in alcoholics and that attenuation in the ligand activation of D2 receptors drives craving and relapse of alcoholism (Volkow et al., 2013; Feltmann et al., 2017)."

6. Line 175 – It is unclear why these experiments were done. Is dopamine known to control pharyngeal pumping? Is dop-2 expressed in the pharyngeal circuitry? Also, the conclusions based on these experiments seem a bit broad. What about other muscles?

Thank you for this comment, we have reworded this section and also added egg – laying experiments to this section. The changes and updates are indicated in Figure S3 and lines 193-205.

7. Line 184-185 (and 497) – standard C. elegans terminology is not used properly. dop-2p:dop-2:cfp

We have corrected the terminology (lines 209, 553 and 569)

8. Line 201 – "defective signaling"? Isn't it enhanced dopamine release?

We have replaced defective with enhanced (line 225)

9. Line 201 – The authors state they were unable to find a PDE specific promoter but then use a strain later with the asic-1 promoter. Rescue experiments could be done using this promoter.

The *asic-1* promoter shows expression in all dopaminergic neurons, it is not PDE specific. In the posterior region since there is just one pair of dopaminergic neurons, it was used to mark the two PDE neurons. Using this promoter, we would not be performing rescue experiments that are different from *dop-2* promoter rescue experiments. However, in order to further assess *dop-2* mutant rescue, we have used the *gpa-14* promoter (expressed in multiple neurons of the animal including the dopaminergic neurons ADE), which does not rescue the *dop-2* mutant phenotype in EtOH. Another promoter, *gpa-16* (expressed in multiple neurons including the posterior neuron PDE, but not the head dopaminergic neurons) significantly rescues the posterior body bends defects seen in *dop-2* mutants on EtOH. This data is shown in Figure S4 and detailed in lines 225-236.

10. Line 215 (Typo – DA neuron) and also is DOP-2 functioning through DVA? Or, is DOP-2 functioning to modulate dopamine from PDE to DVA? This should be reworded.

Thank you for this comment. We have reworded this sentence (lines 249-250).

11. Lines 215-232 – It is very difficult to understand the rationale and conclusions here. For example, Line 227 – Removing dopamine suppresses EIS would seem more of an accurate summary of the results. Also, the conclusion on lines 231-232 are hard to follow. It is unclear what is meant by cat-2 functioning through dop-2?

We have made changes to the lines indicates (lines 253-254, 261-262 and 264-265).

12. Line 324 – more accurate to say nlp-12(OE)

Thank you for this comment. We have changed the overexpression (++) symbol to OE throughout the manuscript.

13. Line 389 – typo "dopamine synthesis mutant"

We have changed this to dopamine biosynthetic pathway mutant (line 438).

14. Line 390 – more accurate to say acr-16(OE)

Thank you, we have made this change (also see point 12 above).

Reviewer #2

In this paper, Pandey et al identify a role of the dopamine receptor DOP-2 in regulating ethanol-dependent behaviours in C. elegans. The authors begin by testing mutants of the dopaminergic pathway in an established assay for ethanol-dependent behaviours, and find that dop-2 mutants display an ethanol-induced sedative (EIS) behaviour after prolonged ethanol exposure. After rescuing this phenotype by re-expressing dop-2 from its endogenous promoter, they ablate the posterior dopaminergic neuron PDE and find that it is required for the EIS behaviour of dop-2 mutants. Using genetic interventions, dopamine supplementation and FRAP as readout for synaptic vesicle fusion rate, they show that EtOH exposure results in increased dopamine neurotransmission from PDE synapses in dop-2 mutants. Further, the authors build on previous work, identifying DOP-1 and neuropeptidergic signalling from DVA as regulators of locomotion, to further characterize the mechanism that underpins ethanol-induced sedative behaviour in dop-2 mutants.

The experiments were performed thoroughly, and the evidence presented seems convincing (with a few exceptions, noted as major revision below). However, there are a number of issues throughout the manuscript that make the data difficult to interpret, and there are some aspects of the proposed mechanism that need to be clarified.

We are happy that the Reviewer thought that our experiments were thorough and the evidence presented was largely convincing. We thank the Reviewer for their comments that we have responded to below. We have also tried to clarify aspects of our data interpretation and the proposed mechanism.

Major comments:

- The authors used laser ablation to determine the cellular focus of dop-2 in EIS behaviour, by ablating PDE neurons in wild type and dop-2 mutants. Although this experiment shows that PDE neurons are required for the EIS phenotype of dop-2 mutants, it does not proof that DOP-2 is required in PDE. An alternative explanation might be that dop-2 indirectly affects PDE function. The authors conclude that dop-2 functions in PDE neurons to regulate EIS behaviour, but additional evidence is

needed to show that dop-2 indeed acts in PDE. A conditional system using promoters that uniquely overlap in PDE could be used when cell-specific promoters are not available.

We thank the Reviewer for this comment. Unfortunately we were unable to perform rescue using a conditional system of overlapping promoters. However, to get further insight into where DOP-2 may be functioning to rescue the *dop-2* dependent EIS phenotype we have performed the following additional experiments:

- We have used the *gpa-14* promoter (expressed in multiple neurons of the animal including the dopaminergic neurons ADE but not the posterior PDE neuron). This promoter expressing DOP-2 did not rescue the *dop-2* mutant phenotype in EtOH.
- We also performed the rescue experiment using the *gpa-16* promoter (expressed in multiple neurons including the posterior neuron PDE but not the anterior dopaminergic neurons) largely rescued the posterior body bends phenotype seen in *dop-2* mutants on EtOH.

  These data have been added to Figure S4 and lines 225-236.

- In the circuit model (Figure 7), the authors depict DOP-2 as an autoreceptor that regulates dopamine release in PDE, but they do not discuss the possibility that DOP-2 may act in additional neurons. DOP-2 is expressed in several other neurons where it may (also) indirectly affect PDE function. This should be clarified in the model and discussion, since the authors did not test a putative role of dop-2 in other neurons. Furthermore, additional evidence is needed to show that dop-2 is required in PDE for ethanol-dependent behaviours.

Thank you for this comment. We consistently saw that the EIS phenotype was more prominent in the posterior region of the animal. In this region, the PDE neurons are the only dopaminergic neuron. Hence, our model indicates the PDE neurons. We have performed more rescue experiments indicated in the point above. We have added the line “However, our data does not preclude the possibility that other dopaminergic neurons could also be involved in this process. These neurons may function through as yet unidentified mechanisms to allow for the EIS phenotype seen in *dop-2* mutant animals” to the manuscript (lines 531-534).

- Figures 2d-e and 4a-b show that PDE neurons are required for EIS behaviour in dop-2 mutants and have increased dopamine neurotransmission. Because dopamine is secreted, is it possible that other dopaminergic neurons show enhanced dopamine release and contribute to the EIS behaviour of dop-2 mutants as well?

Thank you for this comment. Since, we consistently saw that the EIS phenotype was more prominent in the posterior region of the animal. In this region, the PDE neurons are the only dopaminergic neurons. Hence, our model indicates the PDE neurons. We have performed more rescue experiments indicated in the first major comment. However, we do agree with your comment and in the discussion section we have

added the line “However, our data does not preclude the possibility that other dopaminergic neurons could also be involved in this process. These neurons may function through as yet unidentified mechanisms to allow for the EIS phenotype seen in *dop*-2 mutant animals” (lines 531-534).

- The figure panels do not show all statistical comparisons and often it is not clear which genotypes/conditions have been compared, making it difficult to interpret the data and assess whether the results support the main conclusions. All statistical comparisons should be included in the figure panels. For each p value, figure panels should clearly depict which genotypes/conditions were compared.

Thank you for this comment. We have added statistical comparison to all the figures and have elaborated this in the figure legends as well.

- The authors made several claims based on double mutant analyses (e.g. in Figure 3, 5 and 6), but only show statistical comparisons to wild type and not to the single mutants. Statistical comparisons of single and double mutants should be included to assess whether the dop-2 phenotype is mimicked or suppressed and whether genes function in the same genetic pathway or not.

We have now added the statistical comparisons with *dop*-2 mutants to Figure 3, 5 and 6.

- Also, in overexpression and supplementation experiments (Figures 3c-d, 5e-f, 6a-b, 6e-f), statistical comparisons are only shown for wild type making it difficult to assess if overexpression or supplementation suppresses or mimics the dop-2 phenotype. Statistical comparisons to other genotypes/conditions should be added to support the main conclusions.

We have now added the statistical comparisons with *dop*-2 mutants for figures S4 and S6.

- The authors state that loss of dop-1 function significantly decreases body bending (lines 288-290 and Figure 5a-b). However, this statement contradicts the data depicted in Figure S1-1 where dop-1 mutants did not show significantly altered body bending. The effect of dop-1 in ethanol-dependent behaviour should be clarified. If dop-1 mutants show locomotory defects after prolonged EtOH exposure, do these mutants have defects in the absence of EtOH?

The locomotory behaviour of *dop-1* mutant animals are shown to be affected by exposure to ethanol in our graphs in figure 5. However, we did not observe this defect during the initial screening of the dopaminergic receptor mutants as shown in Figure S1. When we later performed these experiment for the data shown in Figure

5a and b, the strain was outcrossed multiple times in the lab, maybe these conditions led to the difference. Further, unlike the *dop-2* receptor mutants, the phenotype seen with *dop-1* is not obvious when we visualize the animals or from plate phenotypes seen in figure 1g.
The *dop-1* mutants don't show obvious locomotory defects in the absence of ethanol as observed by us and also shown previously (Wang D., Yu Y. *et al, PLoS One*; 2014). We have noted this discrepancy in the supplementary figure legends section (lines 7-10 in supplementary information file).

- Similarly, the initial data on anterior body bending shows a defect in dop-2 mutants (e.g. in Figure 1f). However, this defect is not seen in some of the following experiments (e.g. in Figure 2b and 6d). Can the authors explain where this variability comes from?

Thank you for this comment. There are small differences observed with anterior body bends in *dop-2* mutants in most of the experiments, however significant differences are not observed in all experiments. The assay is very sensitive to the dryness of assay plates and small external movements, it is possible that these conditions gave small differences on different days. The difference in posterior body bends however is very robust. In order to get a better picture of our entire data set, we have pooled all the data for movement of WT and *dop-2* mutants in the presence of ethanol and show this data in Figure S1c and d and discuss this data in lines 13-20 of the supplementary information file.

- Based on DVA-specific rescue of dop-1, the authors conclude that the dop-2; dop-1 animals revert to the dop-2 like EIS behaviour (Figure 5a-b and lines 295-297). In Figure 5a-b, statistical comparisons between the transgenic rescue line and the double and single mutants should be included to support this statement.

Thank you. We have added these statistical comparisons for Figure 5a and 5b.

- Overexpression of nlp-12 has previously been shown to affect basal locomotion and body bending in the absence of EtOH. These effects were found to be mediated by the receptors CKR-1 and CKR-2 (Ramachandran et al., 2020). The data presented in Figure 5g-h suggests that ckr-2 is involved in EIS behaviour of dop-2 mutants. Is this effect specific to CKR-2, or is CKR-1 involved as well?

Thank you for this interesting comment. We have looked at the double mutants between *ckr-1* and *dop-2* and have found that *ckr-1*, unlike *ckr-2,* does not suppress the *dop-2* EIS phenoptype. We have added these results to Figure S6g and h and lines 388-393.

- In Figures 6a-b and 6e-f, aldicarb treatment and acr-16 overexpression reduces body bending in wild-type animals exposed to EtOH. Are these effects specific to EtOH-exposure, or does aldicarb and acr-16 overexpression also affect body

bending in the absence of EtOH? The latter is not shown but could imply that the observed effects are not specific to EIS behaviour. In Figure 6a-b, the cha-1 control strain shows a significant decrease in the frequency of body bending, unlike what the authors state in line 371. The corresponding control condition (cha-1 – EtOH), however, is not included in this figure. The authors should add the necessary controls in the absence of EtOH and aldicarb to figures 6a-b and 6e-f.

Thank you for this suggestion. Yes the *acr-16* overexpression EIS phenotype is specific to the presence of EtOH. Figure S7c and d show that ACR-16 overexpression does not show a phenotype in the absence of EtOH or presence of aldicarb. We have added this data in lines 441-443. We have added the *cha-1*-Aldicarb to Figures 6a and b and also show *cha-1* phenotype in the absence of EtOH with and without aldicarb in figure S7c and d. These data have been added in lines 418-422.

Minor comments:

- While the study appears to be sound, the authors should revise the language to improve readability and more clearly explain the rationale for the experimental work.

We have tried to revise the language and improve the readability of the manuscript. We have also more clearly explained the rationale for each experiment.

- The authors should carefully cross-check references in the main text and reference list. Some references mentioned in the main text seem to be missing in the reference list, e.g. Prescott and Kendler, 1999 and Schukit and Smith, 1996, Bettinger and Davies, 2014, etc.

We apologise for this and thank you for pointing out this omission. We have carefully gone ove the manuscript to make sure that every reference is available in the bibliography.

- Several citations in the text are not formatted correctly, for example, line 346-347 "Shankar Ramachandran and Christopher M. Lambert, 2020)

Thank you for this. We have made the appropriate changes to this citation (lines 352-353 and 361).

- Individual data points in box plots are difficult to distinguish and would be clearer when plotted with smaller symbols.

Thank you for this suggestion, we have used smaller dots for all plots.

- Throughout the manuscript, the main post-hoc test is annotated as Turkey-Kramer multiple comparison test. Do the authors refer to the Tukey-Kramer multiple

comparison test?

We are very sorry for this mistake. It is Tukey-Kramer multiple comparison test. Thank you for pointing out this error.

- In Figures 2a-b, transgenes for rescue of dop-2 should be noted in italic, i.e. Pdop-2::dop-2. Likewise, in figure 5e-f, PDVA::nlp-12++ should be italic (not capitalized).

Thank you, we have made these changes.

- Figure 6a-b, writing aldicarb instead of the abbreviation "A" would be clearer.

Since space is limited in each figure, we have used "A" instead of aldicarb. We have expanded this in the figure legends.

- Lines 44-46 and 62-66: Replace or remove "enhanced functioning of the DVA interneuron", since DVA activity or output was not tested in dop-2 mutants. Similarly, the authors cannot claim that DVA releases NLP-12 resulting in the excitation of cholinergic motor neurons, because NLP-12 release and motor neuron activity was not tested after ethanol exposure.

Thank you, we have made these changes.

- Line 86: change "synaptic molecules" because not all indicated examples have been located at synapses.

Thank you, we have made this change (line 85).

- Lines 140-143: Recent work has shown a role of DOP-2 autoreceptors as modulators of synaptic vesicle fusion in dopaminergic neurons (Formisano et al., 2020). Thus, dop-2 mutants have known defects associated with dopamine signalling. This work is relevant to the authors' findings and should be referenced and discussed.

Thank you for this comment and we apologise for this oversight on our part. We have now referenced and discussed the work by Formisano et al., 2020 (lines 484-485).

- Lines 146-154: The authors refer to a previous study by Davies and McIntire that studied the effects of 400 mM ethanol exposure on body bending. They state that similar phenotypes were observed for wild type animals in their study. However, in Figure 1, body bending was only quantified at 120 min after ethanol exposure. Short-

term effects prior to this time point are not shown. The authors mention that dop-2 mutants behaved similar to wild type animals at earlier time points and are thus defective in recovery upon prolonged EtOH exposure. However, this data is not shown in the manuscript. The observation that dop-2 mutants show normal ethanol-induced behaviours upon short-term EtOH exposure is relevant to interpret the dop-2 phenotype (and to compare data in this study with previous work) and should be included in the manuscript.

Thank you for this comment. We have now performed a timecourse of WT and *dop-2* mutant animals behaving in EtOH at 10 min, 30 min, 60 min, 120 min, 300 min and overnight. These data have been added to the manuscript in lines 177-186 in the results section, lines 589-594 in the methods section and in Figure S2.

- Line 158-161: The authors write that they "did not find any readily observable locomotory defects in other mutants in the dopamine pathway". This contradicts the data in Figure S1-1, which shows significant phenotypes for cat-2 and dop-3 mutants. Mutants of slo-1 control animals also do not appear "insensitive to EtOH" based on the data in Figure S1-1.

By observable defects we meant by just looking at the animals and the tracks they made on the plates. We realise that this is confusing and have discussed this further in lines 163-172. In case of *dop-2* the tracks we see on the plates are very distinct circular tracks. We have also added these images to Figure 1g.

- In Figure 1, the data in panels e and f cannot be interpreted without the results of the experiment in absence ethanol (depicted in panels g and h). Merge data in panels e and g and panels f and h, or include the figure panels without ethanol in e and f.

Thank you for this suggestion. We have now merged the data in the previous panels of Figure 1 e and g as well as f and h. This data is now shown as Figure 1 e and f.

- In Figure 1, the data for wild-type animals (-EtOH) in panel c seems identical to the wild type data presented in panel g. Likewise, the data for wild-type animals (+ EtOH) in panel c seems identical to the data presented for wild type in panel e. Data for panels c, e and g should be merged or it should be indicated that the same wild type dataset was used in these figure panels. In addition, the figure legend of Figure 1 states that the same videos were used to analyse body bends/1 min (panels c/e/g) and amplitude (panels d/f/h), but the wild type data in panel d does not match the wild type data in panels f and h (as is the case for body bend/ 1 min). Is this an error, or were different videos used for analysing body bends/min and amplitude?

Thank you for this comment and we apologise for this confusion. Yes, the data sets used for panel c are identical to panel g, for both +/- EtOH conditions in WT. In order to remove any confusion we have merged the figures as suggested (e and g and f and h). Yes, the same videos had been used for quantitation of body bends, but a few different videos were added while quantitating the amplitude, we have removed

those videos and now use the same data sets for both body bends and amplitude. This will allow for better comparisons. The updated data is shown in Figure 1d and f.

- Line 185: states that transgenic rescue lines for dop-2 expressed a Pdop-2::dop-2::cfp construct, whereas a Pdop-2::dop-2::mCherry construct is mentioned in the methods section.

Thank you for pointing this out. This was an error on our part. It was the P*dop-2::dop-2::cfp* construct, we have corrected this (line 553).

- Figure 2d-e: Do PDE-ablated dop-2 mutants show a significantly higher frequency and amplitude in body bending when compared to mock-ablated dop-2 mutants?

Thank you for this comment. Yes, *dop-2* mutants show significantly higher frequency and anplitide of body bends when compared to mock ablated *dop-2* mutants. We have indicated the significance in Figure 2d and e.

- Lines 229-231: state that cat-2; dop-2 mutants show "similar behaviour as was seen in cat-2 mutants [...] These results indicated that cat-2 could be functioning through dop-2 [...]". This statement is not supported by the data in Figure 3a-b, as cat-2 single mutants show increased body bending, whereas cat-2; dop-2 double mutants do not. The cat-2 mutation seems to suppress the dop-2 loss-of-function phenotype, although statistical comparisons to the single mutants should be included to support this conclusion. Furthermore, it is unclear from the presented data if cat-2 functions through (i.e. upstream) of dop-2. If dop-2 functions as an autoreceptor in PDE neurons, cat-2 may also be regulated by dop-2 function. This statement should be corrected, and statistical comparisons to the cat-2 and dop-2 single mutants should be included in Figure 3a-b.

Thank you for this comment, we have revised the text (lines 264-265) and added the statistical comparison for Figure 3a and b.

- Line 253: Figure S3 is missing in the supplementary information.

Thank you and we apologise for this oversight. We have changed the numbering of supplementary figures, which now read from Figure S1- S7 (total 7 supplementary figures). We have also gone over the text and cited them appropriately.

- Figure 4b: Fluorescence recovery in wild type animals and dop-2 mutants (-EtOH) is fast at the start of the experiment and then quickly reaches a plateau around 20% recovery. This percentage is low compared to the work of Hardaway et al (2015) using the same reporter strain, where wild type animals showed recovery up to 80%

in 2 minutes. Could the synapse have been damaged by photo-bleaching? Can the authors explain this difference in % recovery?

In the work by Hardaway et al (2015) the PDE processes were bleached to an intensity 20–30% that of the original fluorescence value, while we have bleached to an intensity of 10-15% of the original fluorescence value, the difference in percentage of recovery observed maybe due to the difference in the bleaching intensities.

- Line 263-264: References supporting a role of D2-like receptors in DAT-1 localization are missing.

Thank you. We have now added the required reference (line 298).

- The authors nicely show that loss of nlp-12 or ckr-2 suppresses the dop-2 phenotype after prolonged EtOH exposure, and that this phenotype is recapitulated by nlp-12 overexpression. Do nlp-12 and ckr-2 act in the same genetic pathway underlying EIS behaviour in dop-2 mutants? Testing nlp-12; ckr-2 double mutants or nlp-12(++); ckr-2 animals for EIS behaviour could further strengthen this notion and support the ligand-receptor interaction suggested in the circuit model (Figure 7).

Thank you very much for this suggestion. We have performed this experiment and find that *ckr-2* mutants supress the *nlp-12(++)* EIS phenotype. We have shown this data in Figures S6e and f and described this experiment in lines 384-387.

- Is it known if EtOH exposure or increased dopamine release from PDE increases NLP-12 release from DVA? The authors include this statement in the final conclusion (lines 474-480) but did not quantify NLP-12 release in this study.

Thank you for this comment. We have modified this statement (lines 531-536).

- Figure 6e-f: indicate the promoter used for overexpression of acr-16 in figure 6e-f.

Thank you, we have done this in figures 6e-f.

- Line 419: states that this study shows for the first time the role of chronic EtOH treatment for extended periods of time up to 24 hours. All data presented in the study have been collected at 120 min of EtOH exposure which does not support this statement.

We have now performed a timecourse of WT and *dop-2* mutant animals behaving in EtOH at 10 min, 30 min, 60 min, 120 min, 300 min and overnight (16 hours). This data is discussed in lines 177-186 in the results section, lines 589-594 in the methods section and in Figure S2. We have also changed the statement to extended periods of time up to 16 hours (line 470).

- Line 434-435: The conclusion that DAT-1 surface transport is dependent on DOP-2 is too strong. The authors only quantified DAT-1::GFP levels in dop-2 mutants but did not perform additional experiments, such as rescue of the DAT-1 transport phenotype, to show dependency on dop-2.

We have modifed this statement (lines 487-488).

- Line 490: include the allele of ckr-2 used in this study (LSC32 is the strain name).

Thank you. We have added the allele of *ckr-2* used in this study (line 546).

Reviewer #3

Increased dopaminergic neurotransmission results in ethanol dependent sedative behaviors in Caenorhabditis elegans
This is an interesting study describing a role for dopamine signalling in ethanol-induced behaviours using the model organism Caenorhabditis elegans. A role for the dopamine autoreceptor-encoding gene, dop-2, in the response to alcohol was identified. In addition, the study also reports a role for the posterior dopaminergic sensory neuron (PDE) in conveying this behaviour, and specifically shows that the exposure of dop-2 mutants to ethanol results in enhanced secretion of dopamine from PDE which stimulate the DVA interneuron to release a neuropeptide, NLP-12. The authors report that this then leads to excitation of the cholinergic motor neuron which impacts worm movement.

The authors employ a range of techniques, including behavioural bioassays, gene localisation tool, neuronal ablation and FRAP, to generate the data presented. The experiments appear to be robust and provide data that support the conclusions. This study provides interesting data for especially for researchers focussed on the dopaminergic pathway and the impact of alcohol on neurosignalling and behaviour. The manuscript is very well-written and the figures are clear.

Overall this paper presents useful data that should be communicated to researchers working in this area. In my opinion it would be suitable for publication in PLOS Genetics, and represents progress in the discipline.

We thank the Reviewer for their comments and appreciation. We are happy that they feel that our manuscript is suitable for publication in *PLoS Genetics*. Below we have responded to all their comments.

General comments:

In my opinion no further experiments are essential for publication however a few additional experiments may provide interesting data that would aid further unravelling

of the signaling system involved - the authors may wish to follow these up in future.
1. The authors describe the impact of ethanol on the body wall and pharyngeal muscle systems in the dop-2 mutant where they found an effect on body wall muscle (measuring locomotion) and no effect on pharyngeal muscle (measuring pumping), relative to WT worms. I wonder if the authors considered looking at the impact on reproductive muscle activity – using egg output as a measure of reproductive muscle function? This would have been interesting as the reproductive muscle is another major muscle system in nematodes, and the egg output assays are well-established for C. elegans.

Thank you for this useful suggestion. We have gone ahead and performed the egg-laying assays with WT and *dop-2* mutant animals. Our results reveal that in the presence of 400 mM EtOH there was significant defect in the *dop-2* mutants in the number of eggs laid after 16 hrs when compared to WT animals, indicating that reproductive muscles are probably affected along with the posterior body wall muscles. We have added this data to Figure S3a and b and lines 193-201.

2. The authors link the posterior DA neuron – PDE – to the phenotype observed on exposure of the dop-2 mutants to ethanol, and provide rationale for this approach, however there are additional DA neurons that have been identified in C. elegans (in addition to PDE) and I would have liked to have seem similar experiments for these neurons to determine if they also have a role in the observed effects? These cannot be ruled out.

Thank you for this comment. DOP-2 expression is also found in PDA, PLM, PVC and FLP neurons in addition to PDE in the posterior region of *C. elegans*. However, DVA shows strong chemical synapses with PDE (61) as compared the other neurons; PDA (-), PLM (5), PVC (8), FLP (3), hence we carried our experiments using PDE. We agree that effects from other neurons cannot be ruled out.

3. Further to above, the authors focus on NLP-12 which is released from the DVA interneuron to enhance ACh release at neuromuscular junctions (previously established) – and they link this to the EIS phenotype (novel here). Does DVA contain any additional neuropeptides that may also be tentatively linked with the observed phenotype?

We have not come across studies reporting any other neuropeptides being secreted from the DVA interneuron. However this will be an interesting follow-up to this work. Thank you.

Minor comments:
I would like to make some minor comments on the manuscript which the authors may wish to consider.

Abstract and Author Summary:
It is my opinion that the abstract and author summary are well written and accurately reflect the content of the manuscript and the key outcomes of the research.

Thank you.

Introduction:
Generally, the introduction is quite well written and broadly covers the background detail required. Some minor considerations identified below.

Line 84
Replace 'functions' with 'function' or 'function(s)'

We have made this change (line 85).

Line 89-90
Replace……'which in turn induces' with 'which, in turn, induces'

Thank you, we have made this change (line 89).

Line 95
Replace: have also been identified, these receptors are thought to regulate the release of DA……with have also been identified; these receptors are thought to regulate the release of DA

Thank you, we have made this change (line 97).

Line 104 and again in Line 108, for example, species names should be written in full at the beginning of a sentence?

We have made this change throughout the manuscript (lines 110, 115 and 627). Thank you for this input.

Line 109
Replace DA receptors, like their mammalian counterparts also belong to two subfamilies……with DA receptors, like their mammalian counterparts, also belong to two subfamilies…….

We have made this change (line 116).

Line 110
In relation to the statement: EtOH shows its effect in a concentration dependent manner, acting as stimulant at lower concentration and depressant at higher concentration, please clarify if this is the effect on the receptor? Or another

behavioural attribute? This is not clear and the statement appears to contradict the next sentence beginning…..'Studies in C. elegans have reported….'
The authors communicate that they developed an EtOH dependent assay but it is not clear if this is a novel assay? I understand that the phenotype observed on chronic exposure is novel?

Thank you for this comment. We have removed the sentence "EtOH shows its effect in a concentration dependent manner, acting as stimulant at lower concentration and depressant at higher concentration". Since multiple Reviewers had problem with the phenotype being called novel, hence we have removed the word "novel" from the manuscript. We agree that the assay has been previously developed and have cited the original paper multiple times.

Line 119
Replace 'Mutants in the D2-like autoreceptor, dop-2 displayed a novel locomotory phenotype when exposed to 400 mM EtOH' with 'Mutants in the D2-like autoreceptor, dop-2, displayed a novel locomotory phenotype when exposed to 400 mM EtOH'

We have made this change (line 125).

Results and Discussion:

The results are very well described and figures accurately reflect the content.

Thank you very much for appreciating this work.

Line 147
Ethanol (EtOH) already introduced in line 145 so any abbreviations should come at the first introduction.

Thank you, we have removed Ethanol from line 152 and added the abbreviation EtOH to line 150.

Line 158
'We did not find any readily observable locomotory defects in other mutants in the dopamine pathway.' – please clarify at this point which dopamine pathway mutants were examined.

By observable defects we meant by just looking at the animals and the tracks they made on the plates. We realise that this is confusing and have discussed this further in lines 163-166. In case of *dop-2* the tracks we see on the plates are very distinct circular tracks. We have also added these images to Figure 1g.

Line 197 Bhattacharya et al should read Bhattacharya et al (2014) – also not sure that this should be communicated as a 'recent' publication…?

Thank you. We have added Bhattacharya et al (2014) to line 221 and removed recently from line 222.

Line 419
Insert space between up and to

Thank you, we have added the space between up and to (line 470).

Line 466
Drosophila melanogaster should be abbreviated as spp. mentioned before.

Thank you, we have done this (lines 508 and 519).
