# Supplementary material for: Increased dopaminergic neurotransmission results in ethanol dependent sedative behaviors in Caenorhabditis elegans
Source: PLoS Genet. 2021 Feb 1;17(2):e1009346. doi: 10.1371/journal.pgen.1009346 (PMC7877767; doi:10.1371/journal.pgen.1009346)
Supplement: Attachment — Submitted filename: Pandey, Singh et al Response to Reviewer comments Dec 2020.pdf [file pgen.1009346.s021.pdf]

Overview:

Enclosed with this letter you will find our manuscript entitled “Increased dopaminergic neurotransmission results in ethanol dependent sedative behaviors in *Caenorhabditis elegans"* that we had submitted for publication to *PLoS Genetics* as a Research Article (Manuscript no. PGENETICS-D-20-00998).

We thank the Associate Editor, Liliane Schoofs, the Editor-in-chief, Gregory P. Copenhaver and the Reviewers’ for their time and comments and we have tried our best to respond to the points raised by them.

Our responses to the Reviewers’ comments are given below and the revised version of the manuscript is enclosed in this resubmission. The response to each comment and the changes made to the manuscript are indicated in blue. We have gone through the comments carefully, and are now submitting a revised manuscript that we believe takes into consideration all the concerns of the Reviewer. We hope that the Editor and Reviewer agree that these changes have addressed their concerns and that our manuscript is now acceptable for publication in *PLoS Genetics*.

Reviewer #1

Overall, the authors have submitted an improved manuscript but some of my initial concerns remain unsatisfied. However, these are mostly minor concerns.

Thank you. We have tried to respond to all your comments below.

•*The authors have addressed my concern about the novelty of this behavior and it is now clearer how this behavior occurs. I appreciate that they show a more detailed and temporal description of EIS in the manuscript. I still feel that the manuscript would benefit from a more detailed description, possible graphically in a main figure of the text. The additions to the supplement are nice but they seems slightly hidden. It is not clear to the average reader how EIS is unique from ethanol-induced paralysis. Couldn’t the back of the worm be paralyzed longer and that EIS is actually just a decoupling of the paralysis between the front and back? This was really the concern I had initially. It would help if the authors clarified this in the manuscript. Maybe ethanol-induced paralysis in dop-2 mutants was simply a paradigm for revealing a function for DOP-2.*

We thank the Reviewer for this comment. In order to better allow the reader to understand the assay we have added the time-line of paralysis and recovery as figure 1a. We have deleted Figs 1c and d as the data from Figs. 1c and d was present is Figs 1e and f (these are now Figs. 1 d and e). We also agree that more details on how the assay is performed will be useful to the reader and helpful for performing the assay, to this end we will send a detailed protocol along with required graphics for publication in *protocols.io* or *bio-protocol* as soon as the manuscript is accepted. We also agree that the EIS phenotype could just be the decoupling of

paralysis of the front and back of the *C. elegans*. The Reviewer is correct in that we have used this phenotype as a paradigm for revealing the function of DOP-2. We have added this to line 180-181.

•*The authors have satisfactorily shown that EIS is reversible.*

Thank you.

•*The conclusions throughout are better worded but still hard to follow in a number of circumstances, which I have detailed below.*

Thank you for your time in helping us improve the text of our manuscript, we have made the changes suggested.

•*The wording in lines 90-96 is still vague and grammatically incorrect. By saying dopamine receptors are GPCRs, then their participation with g-protein signaling would be implied. What G-proteins do they signal through based on work in other animals? Why not just say they signal through the subtype Galpha and be specific?*

Thank you for this comment. We have reworded the paragraph as suggested (lines 90-99).

•*With regards to my initial comment about the pharyngeal muscle, the authors have done additional experiments related to DOP-2's role with egg-laying. Interestingly, egg-laying is also suppressed during their EIS behavior. This brings up the question, is egg-laying also restored in the rescued strains? Is this PDE-dependent or are other neurons sensitive to removing dop-2. This may be beyond the scope of the manuscript but would be a nice addition to the overall description of DOP-2's function.*

We thank the Reviewer for this comment and agree that getting a better understanding of DOP-2 function in egg-laying in the presence of EtOH will be interesting. As suggested, this study would take time and be out of the scope for this manuscript. However, we definitely plan to pursue this aspect of DOP-2 function in future and will evaluate the role of PDE in the egg-laying function.

•*Nomenclature for transgenics is still not used properly in the results, methods and figures. For example, lines 553, 559, 569. Using: dop-2p::dop2::cfp would be more consistent with the field.*
(See: https://wormbase.org/about/userguide/nomenclature#81al793kmj4hieg62d5fb0c--10)

Thank you. We have gone through the entire manuscript and changed the nomenclature of transgenics with p (promoter) added after the gene name (ie *dop-2p* and not P*dop-2*). We have made this change to all the figures and tables as well.

•PDE-rescue could still be accomplished with an intersectional approach; however, the authors have explained that there is still a possibility that other neurons are involved. With this stipulation and the new experiments, they have satisfied my concern about PDE's role here.

Thank you.

•Remove "hence" in line 255

Thank you. We have removed hence from line 255.

•The conclusion in line 264-266 is unclear. Why unlikely? Can this be clarified?

Thank you. We have changed "unlikely" to "is not" (line 269).

•Lines 346-348. What do you mean by over-expression? Multi-copy array? Also, in the figure of which this refers to, it is unclear what PDVA::nlp-12 is referring to. Clarification is needed either here or in the methods, because the methods does not explain this either. PDVA is not the correct nomenclature.

Thank you for pointing this out. We have now added "To study the effect of DVA specific rescue or NLP-12 overexpression (OE), the *nlp-12* promoter was utilized to express DOP-1 or NLP-12 specifically in the DVA interneuron. The transgenic lines for these experiments are denoted as *DVAp*::*dop-1 and DVAp*::*nlp-12*" to the methods section (lines 578-581).

•Line 373 – the nomenclature/grammar is not correct. Would be more accurate to say: "NLP-12 peptides signal through their receptor CKR-2." The way it is phrased is suggesting you are talking about proteins. Also, there are multiple NLP-12 peptides (2). In fact, in this entire paragraph the nomenclature jumps back and forth.

Thank you. We have made the required change (line 377) and also changed the nomenclature of genes and proteins for more uniformity in the paragraph.

•Again, the phrase DOP-2 functioning through CKR-1 is stated (line 380 and line 382). It would be more accurate to describe the mechanism. i.e. Mutations in dop-2 increase dopamine, which signals through DOP-1, increasing NLP-12, etc. It seems incorrect to just say DOP-2 functions through CKR-2. Same for lines 392,393.

Thank you. We have made this change in lines 383-385 and have also changed the wording to "upstream" instead of "through" in line 398.

•Line 395 – there are two distinct NLP-12 peptides. Would be more accurate to describe the model in those terms.

Thank you. We have made this change (lines 400-402).

•Lines 426-428 are hard to follow. Also, NLP-12 (upper-case) refers to protein. So, "increased NLP-12 expression" is incorrect.

Thank you for this comment. We have changed the sentence to "increased NLP-12 release…" (430-433).

•Lines 440-441 – Not worded clearly. ACR-16(OE) is allowing for the EIS phenotype through the dopaminergic pathway? SO, ACR-16 is upstream of dopamine?

Thank you. We think ACR-16 is downstream of dopamine and have reworded the sentences (lines 442-446).

•Lines 444-445 – Same comment – conclusion is not worded clearly

We have reworded sentence (lines 446-447).

•Missing the Ach label in fig 7a.

Thank you. We have added the ACh label to Fig. 7a.

Reviewer #2

The authors have performed several additional experiments and clarified the main text and figures to address the reviewer comments. The additional analyses and discussion have substantially improved the quality of the manuscript, which I support for publication. I have no additional comments, except for the following typos:

Thank you for your kind appreciation. We have made the changes to the typos given below.

Line 373: NLP-12 functions through its receptor...

Thank you, we have changes it's to their in line 377.

Line 460: nucleus accumbens

Thank you four pointing out the spelling mistake in “accumbens”, we have rectified this mistake (line 467).

Reviewer #3

The authors have effectively addressed all of the issues raised by this reviewer. The authors have performed significant additional experiments to address the comments of all reviewers and have enhanced the presentation of data and clarity in their interpretation of findings. In general, the readability of the manuscript has also improved.

Thank you very much for your appreciation.
